# Genome-wide DNA methylation and transcriptome analyses reveal the key gene for wool type variation in sheep

**DOI:** 10.1186/s40104-023-00893-6

**Published:** 2023-07-08

**Authors:** Jiankui Wang, Guoying Hua, Ganxian Cai, Yuhao Ma, Xue Yang, Letian Zhang, Rui Li, Jianbin Liu, Qing Ma, Keliang Wu, Yaofeng Zhao, Xuemei Deng

**Affiliations:** 1 State Key Laboratory of Animal Biotech Breeding, China Agricultural University, No. 2 Yuanmingyuan West Rd, Beijing, 100193 People’s Republic of China; 2 Key Laboratory of Animal Genetics, Breeding and Reproduction, Ministry of Agriculture & Beijing Key Laboratory for Animal Genetic Improvement, China Agricultural University, No. 2 Yuanmingyuan West Rd, Beijing, 100193 People’s Republic of China; 3 Jinfeng Animal Husbandry Group Co., Ltd., Chifeng, 024000 China; 4 Lanzhou Institute of Husbandry and Pharmaceutical Sciences, Chinese Academy of Agricultural Sciences, Lanzhou, 730050 China; 5 Animal Science Institute of Ningxia Agriculture and Forestry Academy, Yinchuan, 750002 China

**Keywords:** Primary wool follicle, RNA-seq, *SOSTDC1*, Whole genome bisulfite sequencing

## Abstract

**Background:**

Wool fibers are valuable materials for textile industry. Typical wool fibers are divided into medullated and non-medullated types, with the former generated from primary wool follicles and the latter by either primary or secondary wool follicles. The medullated wool is a common wool type in the ancestors of fine wool sheep before breeding. The fine wool sheep have a non-medullated coat. However, the critical period determining the type of wool follicles is the embryonic stage, which limits the phenotypic observation and variant contrast, making both selection and studies of wool type variation fairly difficult.

**Results:**

During the breeding of a modern fine (MF) wool sheep population with multiple-ovulation and embryo transfer technique, we serendipitously discovered lambs with ancestral-like coarse (ALC) wool. Whole-genome resequencing confirmed ALC wool lambs as a variant type from the MF wool population. We mapped the significantly associated methylation locus on chromosome 4 by using whole genome bisulfite sequencing signals, and in turn identified the *SOSTDC1* gene as exons hypermethylated in ALC wool lambs compare to their half/full sibling MF wool lambs. Transcriptome sequencing found that *SOSTDC1* was expressed dozens of times more in ALC wool lamb skin than that of MF and was at the top of all differentially expressed genes. An analogy with the transcriptome of coarse/fine wool breeds revealed that differentially expressed genes and enriched pathways at postnatal lamb stage in ALC/MF were highly similar to those at the embryonic stage in the former. Further experiments validated that the *SOSTDC1* gene was specifically highly expressed in the nucleus of the dermal papilla of primary wool follicles.

**Conclusion:**

In this study, we conducted genome-wide differential methylation site association analysis on differential wool type trait, and located the only CpG locus that strongly associated with primary wool follicle development. Combined with transcriptome analysis, *SOSTDC1* was identified as the only gene at this locus that was specifically overexpressed in the primary wool follicle stem cells of ALC wool lamb skin. The discovery of this key gene and its epigenetic regulation contributes to understanding the domestication and breeding of fine wool sheep.

**Supplementary Information:**

The online version contains supplementary material available at 10.1186/s40104-023-00893-6.

## Introduction

Sheep are important livestock animals that supply people with meat, milk, fur, and wool fibers. Wool is a valuable natural textile fiber, and fine wool is used in textile clothing. Fine wool sheep was first developed in Iberia and have been bred for over hundreds of years [1]. Merino is the main fine wool sheep breed since the eighteenth century and has spread widely in the world. In general, modern Merino breeds were cultivated by crossing local sheep breeds with imported Merino, such as the France Merino and Germany Merino [2]. Chinese Merino sheep breeds were mainly produced by crossing Tibetan or Mongolian sheep with Soviet Merino, Rambouillet sheep and Australian Merino [3]. The main distinction of fine and coarse wool sheep breeds is that the coarse wool sheep have a large amount of medullated wool, while the key to fine wool sheep breeding is to select with less medullated wool until only non-medullated wool retained [4, 5]. The medullated wool is developed from primary wool follicle, while the non-medullated wool is developed from primary and secondary wool follicle [6]. Thus, the wool type variation should be due to the diversity of wool follicle types, however, the main determining period of wool follicle morphogenesis is at the embryonic stage, where, primary wool follicles concentrate at embryonic d 50–90 and secondary wool follicles at embryonic d 80–100 [7]. Moreover, the epigenome comprising different mechanisms, e.g., DNA methylation, remodeling, histone tail modifications, chromatin microRNAs and long non-coding RNAs, interact with environ-mental factors like nutrition, pathogens, climate to influence the expression profile of genes and the emergence of specific phenotypes [8]. Multi-level interactions between the genome, epigenome and environmental factors might occur. Furthermore, numerous lines of evidence suggest the influence of epigenome variation on health and production [9]. One of the basic activities in domestic animals is the study of genes and proteins related to economic traits and their study at the cellular or chromosomal level [10]. Besides, the RNA-sequencing of tissues from embryonic stages has been performed, it still poses great difficulties for the study of the mechanisms of wool follicle morphogenesis due to limitation of phenotypic observation and variant contrast [11].Wool follicle development is a progressive process. For example, the structure of wool follicle in embryonic stage is different from that after birth [12]. Compared with the newborn period, the wool follicle structure of fur sheep changes after adulthood [13]. A small number of Merino fine wool lambs with medullated wool had also been found, but by adulthood, medulla wool has all taken off, leaving only non-medulla wool. Since the wool type variation changes with time, this implies that it has the effect of epigenetic modification and regulates gene expression when the DNA sequence remains unchanged [14]. Studies had shown that the skin tissues of coarse and fine wool sheep exhibited different levels of m^6^A modification, which was an important epigenetic regulation at the RNA level [15]. DNA methylation also regulates almost every aspect of biological development [16]. Whole-genome bisulfite sequencing (WGBS) was performed widely by using next-generation sequencing on bisulfite treated DNA, distinguishing methylated cytosines from cytosines [17]. WGBS has been used for epigenetic studies of sheep wool traits, for example, a recent study reported the dynamic changes of methylation pattern at different growth stages of Chinese Tan sheep and identified DMGs that were associated with age and fleece growth [14]. Another example was about the function of methylation dynamics in the transition of the Chinese Zhongwei goat curly fleece characteristic, which suggested the importance of epigenetic inheritance in controlling wool development in Zhongwei goat [18]. However, none of these reports were able to propose specific genes that are epigenetically regulated in relation to wool follicle development.

In previous studies, it has been found that the major genes of fine wool in sheep, such as *IRF2BP2*, are considered to be the most influential gene in fine wool sheep [19, 20]. However, almost all of these gene variations are based on genomic variations [21]. Although there are epigenetic studies related to wool development, few key epigenetic regulatory genes have been reported. In this study, we selected three pairs of full/half sib ALCs and MFs from the same family with almost the same genomic background to specifically search for major genes based on methylation differences. However, the key epigenetic regulatory loci associated with wool development was rarely reported.

## Materials and methods

### Animals and samples preparation

The ALC and MF wool lambs were produced by multiple-ovulation and embryo transfer (MOET), and Aohan fine wool sheep, a Merino related breed in China, were used as donors. A total of four ALC wool lambs were discovered from two families (Additional file 1: Fig. S1), of which three female ALC lambs and three female MF lambs from one ram and two ewes were used for subsequent WGS, WGBS and RNA-seq analysis, these lambs were also used for phenotype determination and gene expression analysis of different tissues. Besides, the same growth environment and feed conditions were provided to these lambs. At 30 days of age, the wool fibers at middle-sided dorsal area of each lamb were collected for determination of wool diameter. One square centimeter of middle-sided dorsal skin of these lambs was collected, and immediately frozen in liquid nitrogen for total DNA and RNA extraction and the preparation of frozen section of skin tissue.

### Preparation of frozen skin sections and phenotype determination

ALC and MF wool lamb skin were sectioned at 10 μm by using freezing microtome (CM1900; Leica, Nussloch, Germany), and stained with hematoxylin and eosin staining kit (C0105S, Beyotime, Shanghai, China). Photographs were taken using the microscope (RVL-100-G, ECHO, San Diego, CA, USA). The four ALC lambs and their full/half-sib of MF wool lambs were used for statistical analysis of wool follicle diameter and wool follicle number. Specifically, for each lamb skin section, the diameter and number of primary and secondary wool follicles are measured and counted in 5 randomly selected fields under the microscope (M1, BEINO, Shanghai, China), and the average of these 5 fields is taken as the wool follicle diameter and number of this lamb. Finally, the ratio of secondary to primary wool follicle (*S*/*P*) is used to evaluate the wool quality. Besides, the wool diameter was measured using a microscope projector (M1, BEINO, Shanghai, China). In order to calculate the average fiber diameter and determine the fiber diameter distribution, wool fibers were collected from ten ALC (collected for four consecutive years in embryo transfer experiments) and ten MF wool lambs, and the Y172 fiber slicer (also called Hastelloy slicer) was used to determine wool diameter, each of which included 200 fibers for testing the wool diameter.

### Whole genome re-sequencing

Skin tissues of the three sibling pairs of ALC and MF wool sheep were collected and place it in 75% ethanol for genomic DNA (gDNA) extraction. gDNA was extracted from sheep skin tissues using the TIANamp Tissue/Cell Genomic DNA extraction kit (DP304, TIANGEN, Beijing, China) following the manufacturer’s protocols. For each DNA sample, a whole-genome sequencing library was built using the Illumina TruSeq DNA Sample Preparation kit (Nextera XT, Illumina, Inc, San Diego, CA, USA) with an insert size of ~ 350 bp. The libraries were sequenced on the Illumina HiSeq 2000 platform. The paired-end reads of 100 bp were generated for each fragment. Sequencing depth was 30×. The fastqc software was used to identify the quality of sequencing data (https://www.bioinformatics.babraham.ac.uk/projects/fastqc/). The fastp software was used to conduct the quality control of sequencing data by the default parameters (https://github.com/OpenGene/fastp). Sequencing reads of WGS were aligned to the sheep reference genome (GCA_016772045.1, NCBI) using the BWA-MEM algorithm with the default parameters (http://bio-bwa.sourceforge.net/https://github.com/lh3/bwa). PCR duplicates were removed with the MarkDuplicates module in the Picard Tools package v2.9.0 (http://broadinstitute.github.io/picard/). GATK 4.0 module HaplotypeCaller was used to conduct variant calling, and variant filtering was done using the parameters “QUAL < 30, QD < 2.0, MQ < 40.0” (https://github.com/broadinstitute/gatk). The VCFtools software was used to convert the variant data file from VCF format to Plink format (https://vcftools.github.io/index.html). For quality control filtering, SNPs were removed under the call rates < 90%, minor allele frequency < 0.05, Hardy–Weinberg equilibrium < 0.00001. The phylogenic tree was constructed by the Neighbor-Joining method using phylip software (https://evolution.genetics.washington.edu/phylip.html) and was draw by Figtree software [22], using data from self-tests (Accession: PRJNA760647) and public databases (Accession: PRJNA304478 [23]), five breeds were used in the construction of phylogenetic tree, shown in Additional file 2: Table S1, Hu sheep (public data, *n* = 3), Ujimqin sheep (public data, *n* = 3), Tan sheep (public data, *n* = 3), Tibetan sheep (public data, *n* = 3), Australia Merino sheep (public data, *n* = 6), ALC and MF sheep (self-test data, *n* = 6), Pan (public data, *n* = 3).

### Whole-genome bisulfite sequencing

Whole-genome bisulfite sequencing (WGBS) was performed as previously described [24]. Specifically, Genomic DNA (a full/half-sib family with three coarse and three fine) were isolated from skin tissues using TIANamp Tissue/Cell Genomic DNA extraction kit (DP304, TIANGEN, Beijing, China) following the manufacturer’s protocols. For each DNA sample, a whole-genome sequencing library was built using the Illumina TruSeq DNA Sample Preparation kit (Nextera XT, Illumina, Inc., San Diego, CA, USA) with an insert size of ~ 350 bp. The libraries were sequenced on the Illumina HiSeq 2000 platform. The paired-end reads of 150 bp were generated for each fragment. The raw reads were filtered to remove contaminated reads. Softwares of quality control were trim_galore and fastqc. Clean reads were mapped to the sheep reference genome (GCF_016772045.1_ARS-UI_Ramb_v2.0, NCBI) using Bismark (v0.23.1). In the whole genome BS-Seq procedure, unmethylated cytosines in genomic DNA were converted into thymines after bisulfite treatment and PCR amplification, but methylated cytosines remained unchanged. The bisulfite conversion rate was calculated using Bismark as the percentage of methylated reads in the total number of clean reads. Default parameters were used by the Bismark software. No mismatch was permitted in the “seed” during alignment. The “bismark_methylation_extractor” script packaged with Bismark was used to extract methylation calls. The methylation level at a C site was determined using the procedure referred to Cokus. The methylation status of each cytosine with higher than fivefold coverage was analyzed using the binomial distribution model. At each position, read depth was used to test whether the number of detected cytosines exceeded the number expected because of sequencing error. This analysis generated coverage statistics for methylated cytosine reads and their contexts (CG, CHG and CHH, where H can be A, T, C, respectively). To analyze differentially methylated genes (DMGs) and differentially methylated regions (DMRs), we compared coarse wool group and fine wool group using methylKit (v.0.9.2). The criteria were applied to identify methylation level differences, which was *Q*-value < 0.01. DMGs and DMRs were annotated using GO and KEGG functional databases to infer gene function.

### mRNA-seq

A total of 5 μg of RNA from each sample was used as input material for RNA sample preparation. Ribosomal RNA was removed by using Epicentre Ribo-zero™ rRNA removal kit (Epicentre, Madison, WI, USA), the mRNA sequencing libraries were constructed by using the NEBNext® Ultra™ Directional RNA Library Prep kit for Illumina® (E7420, NEB, Beverly, MA, USA), according to the manufacturer’s instructions. The mRNA libraries were sequenced on Illumina Hiseq 2000 platform. The sheep reference genome and annotation files were obtained from the Ovis genome website ( ftp://ftp.ncbi.nlm.nih.gov/genomes/all/GCF_000298735.2_Oar_v4.0/GCF_000298735.2_Oar_v4.0_genomic.fna.gz). The clean reads were obtained from raw reads by removing poly-N regions, reads containing adapters and low-quality reads (Trimmomatic). The Q20, Q30, and GC contents of the clean reads were calculated. The reference genome index was constructed by using Bowtie2, and clean paired-end reads were aligned to the reference genome by using TopHat. The mapped reads of each sample were assembled using both Cufflinks and Scripture (beta2) with a reference-based approach. We used the PhyloFit program to compute phylogenetic models for conserved and non-conserved regions among species. The Cuffdiff algorithm was used to calculate the fragments per kilobase of exon per million fragments mapped (FPKMs) of mRNAs. Using a model based on the negative binomial distribution, the differentially expressed genes (DEGs) with an adjusted *P*-value (*P-*adjust < 0.05; Benjamini-Hochberg (BH) multiple test correction) between ALC and MF lambs were identified.

### Integrated analysis of RNA-seq between coarse and fine wool sheep of different growth stages

The transcriptome data includes self-test data (Accession: PRJNA760647) and public database data, of which the public database data includes 4 Tan Sheep (Accession: PRJNA182914 [13]), 3 Super Merino and 3 Small Tail Han (Accession: PRJNA378408 [25]), 9 Aohan fine wool sheep (Accession: PRJNA595784 [26]), and 6 Tibetan sheep (Accession: PRJNA421633 [27]). The description of samples was listed in Additional file 3: Table S2. The fastqc software was used to identify the quality of sequencing data. The fastp software was used to conduct the quality control of sequencing data by the default parameters. Sequencing reads of RNA-Seq were aligned to the sheep reference genome (GCF_016772045.1_ARS-UI_Ramb_v2.0, NCBI) using the STAR algorithm with the default parameters. We used SubRead package featureCount (v2.21) for counting reads. Transcripts Per Million (TPM) method was used for normalization of RNA-seq. The *t*-test method for the gene expression differential analysis. The principal component analysis (PCA) was conducted using Transcripts Per Million (TPM) data through “prcomp() function” in R package “stats”.

### Real time quantitative PCR

Three lambs were used to examine the expression profile of the *SOSTDC1* gene among different tissues, including heart, liver, spleen, lung, kidney, muscle, fat and skin. Each tissue sample was a mixed pool of three different parts of that tissue. These lambs were sexually uniform female one-month-old lambs, including one Aohan fine-wool lamb (Fine wool), one Tan lamb (Coarse wool), and one Ujimqin lamb (Coarse wool). One-month-old lambs were used for relative expression analysis of the *SOSTDC1* gene in skin tissue between coarse and fine wool breeds, including 4 ALC lambs (Coarse wool), 4 MF lambs (Fine wool), 4 Tan sheep lambs (Coarse wool), 4 Ujimqin lambs (Coarse wool) and 8 Aohan fine wool lambs (Fine wool). Each skin tissue sample was derived from a mixed pool of three different parts of the scapula. Quantitative real-time PCR (qPCR) primers of *SOSTDC1* gene (accession number: XM_004007767): F: 5´ CCTCCTGCCATTCATTTCTC 3', R: 5' CGAGATGTATTTGGTGGAACG 3'. The β-actin gene (accession number: U39357) is used as a house keeping gene, The primers: 5' CTGTCCCTGTACGCCTCTGG, TTGATGTCACGGACGATTTCC 3'. Total RNA was extracted by trizol (15596026, Invitrogen, Carlsbad, CA, USA). 500 ng RNA was used to synthesize the first strand of cDNA using FastKing RT kit (KR116, TIANGEN, Beijing, China), Reaction system were carried out in a volume of 20 μL with reaction mixtures purchased from TIANGEN Biotech (Beijing) Co., Ltd. (FP205, TIANGEN, Beijing, China). CFX96TM Real-Time System was used for Q-PCR (CFX96, BIO-RAD, Hercules, CA, USA), thermal cycling conditions were as follows: 5 min at 95 °C; 40 cycles at 95, 55, and 72 °C for 1 min each; and finally 3 min at 72 °C. The results of *SOSTDC1* expression related *β-actin* were test in triplicate.

### Western-blot

One-month-old ALC, Tan, Ujimqin, MF and Aohan fine wool lambs were used for total protein extraction and subsequent Western-blot. In these five lamb breeds, 3 of each were selected for mixing pools, where Merino-1 and Merino-2 were derived from mixing pools with 3 Aohan fine wool lambs skin tissue, respectively. The skin tissues of these lambs were well ground in liquid nitrogen and the total proteins were extracted by IP lysate (P0013, Beyotime, Shanghai, China). The total proteins and the pre-stained protein molecular weight marker (MF291-01, Mei5bio, Beijing, China) were separated by 10% SDS-PAGE and transferred onto nitrocellulose membrane, then blocked one hour with blocking solution (P0252, Beyotime, Shanghai, China). The membrane was incubated overnight with mixed antibodies, that is SOSTDC1 (PK90017S, Abmart, Shanghai, China) antibody with 1:1,000 dilution and β-actin antibody with 1:2,000 dilution (AF5003, Beyotime, Shanghai, China). The overnight membrane was washed three times with washing solution (P0023C, Beyotime, Shanghai, China), then the membrane was incubated with a 1:2,000 dilution of HRP conjugated anti-rabbit secondary antibody (A0208, Beyotime, Shanghai, China) for 1 h at room temperature. Target protein were detected by Gel Documentation System (G:box, SynGene, Cambridge, UK). Band density was analyzed using image J software.

### Immunofluorescence

For immunostaining, frozen sections of skin tissues were fixed with acetone for 30 min and permeabilized with 0.1% Triton-X100 for 5 min. Frozen sections were blocked with 5% BSA for 60 min and incubated with anti-SOSTDC1 (PK90017S, Abmart, Shanghai, China) antibody (1:150) at 4 °C. On the second day, frozen sections were incubated with Cy3-labeled goat anti-rabbit IgG (A0516, Beyotime, Shanghai, China) (1:300) for 60 min in the dark. Nuclei were stained with DAPI (C1005, Beyotime, Shanghai, China). The photographs were taken using a fluorescence microscope (RVL-100-G, ECHO, San Diego, CA, USA).

### Statistical analysis

Differences between two groups were tested using independence Student’s *t*-test, two-tailed *t*-tests were performed with the following *P*-values: ^*^P < 0.05; ^**^P < 0.01; ^***^P < 0.001.

## Results

### Phenotypic comparison of ALC and MF wool lambs

**Fig. 1:**
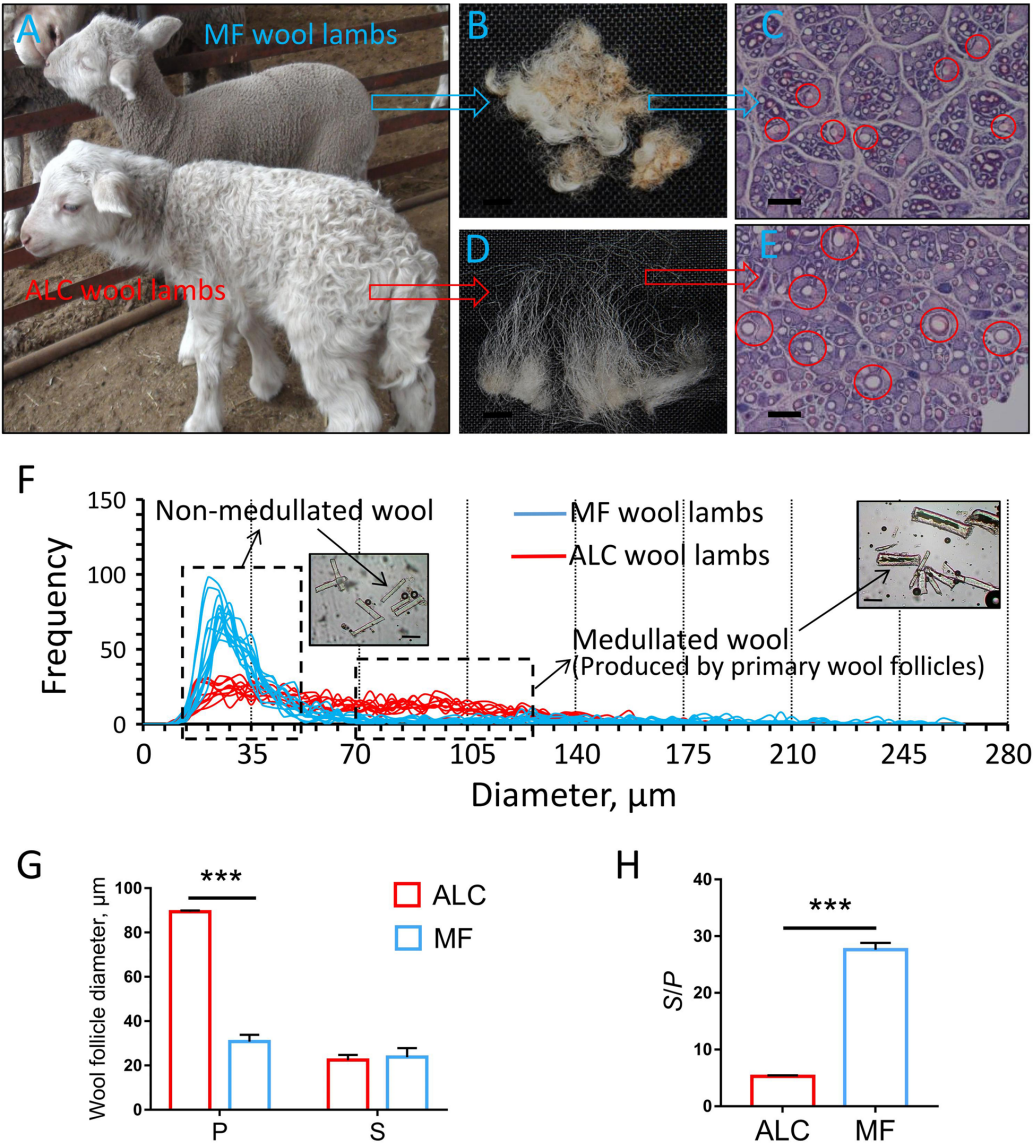
Discovery of ALC wool lambs in fine wool sheep population. **A** The appearance of ALC and MF wool lambs in P30. **B** The wool fibers of MF wool lambs. **C** The Cross-sectioning of MF lamb skin and subsequent HE staining. **D** The wool fibers of ALC wool lambs. **E** The Cross-sectioning of ALC lamb skin and subsequent HE staining. The bars in **C** and **E** are 180 μm. **F** The distribution of ALC and MF wool diameters. **G** Comparison and analysis of primary and secondary wool follicle diameters in ALC and MF wool type lambs. P: primary wool follicles, S: secondary wool follicles. ^***^P < 0.001. **H** Comparative analysis of S/P ratio for ALC and MF wool type lambs. *S*/*P*: Ratio of the number of secondary wool follicles to primary wool follicles. ^***^P < 0.001

During four consecutive years of MOET breeding of Aohan fine wool sheep, We continually find that ALC wool lambs up to one month of age have medullated wool all over their bodies, with a total percentage of 5% (Fig. 1A and Fig. S1). The coat of ALC wool lamb was composed of both medullated and non-medullated wool fibers (Fig. 1D), whereas of MF lambs had only non-medullated wool fibers (Fig. 1B). In addition, both ALC and MF wool lambs consisted of primary and secondary wool follicles, with the difference that ALC wool lambs has larger primary wool follicles than MF wool lambs (Fig. 1C and E). Moreover, the microstructure and diameter distribution of wool fibers also showed that ALC wool lambs contained a large proportion of medullated wool, while MF lambs had non-medullated wool fibers with uniform diameter (Fig. 1F). Given that medullated wool are developed from primary wool follicles [28], we measured wool follicle diameters and *S*/*P* ratio in ALC and MF wool lambs and the results showed that the primary wool follicles diameter was significantly larger in ALC group than that of MF group, while the secondary wool follicles diameter was not significantly different between this two groups (Fig. 1G). In addition, the *S*/*P* tatio of the MF wool lambs were significantly higher than that of the ALC group (Fig. 1H). these results suggested that the postpone development of primary wool follicles should have contribution to the appearance of ALC fibers in the fine wool lamb population.

### WGBS revealed *SOSTDC1* as the key gene associated with the ALC wool type

**Fig. 2:**
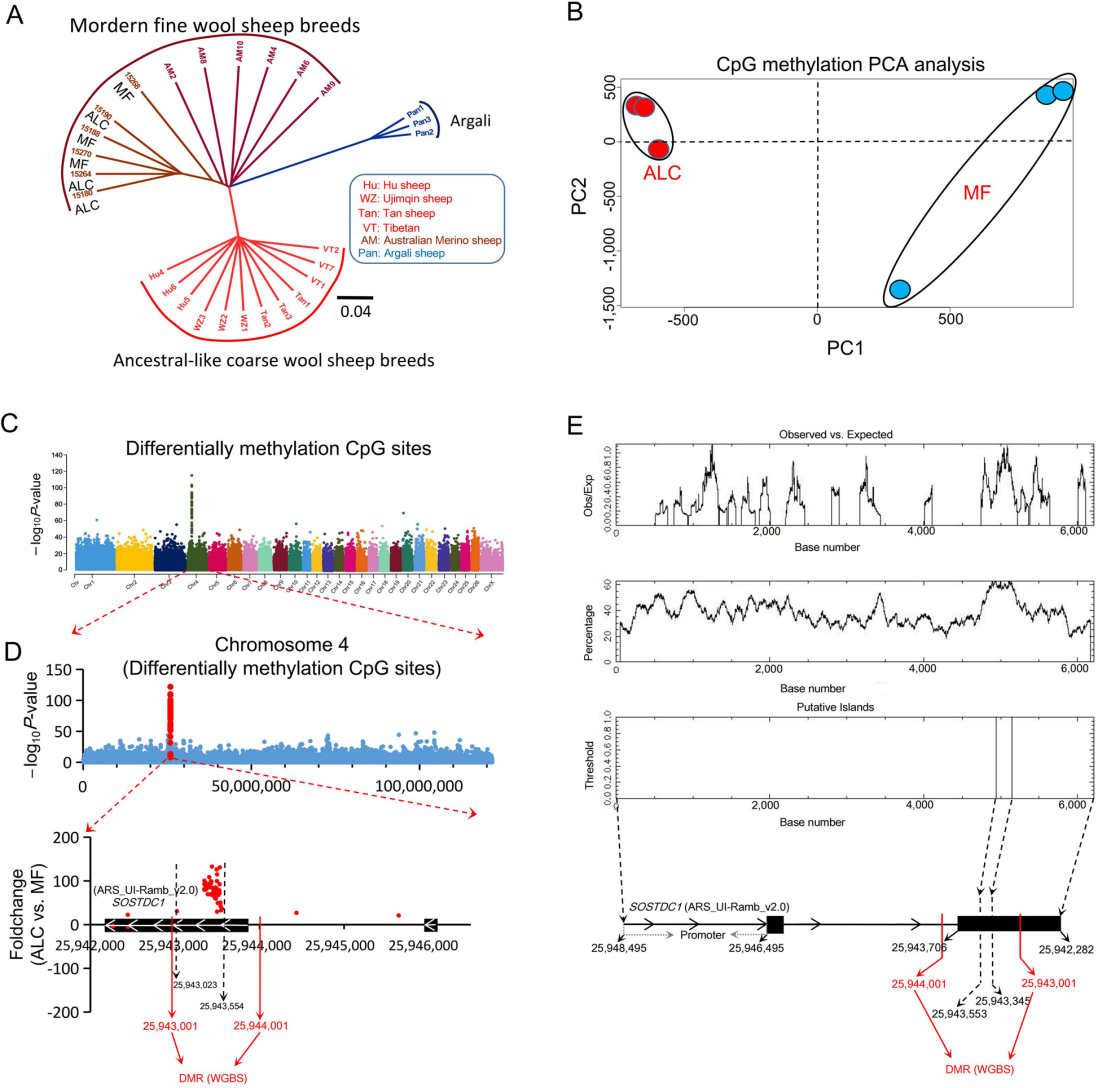
The key gene was revealed by whole genome methylation sequencing (WGBS). **A** The construction of phylogenic tree among different sheep breeds according to whole genome resequencing data. **B** The principal component analysis (PCA) of WGBS data in ALC and MF group. **C** Manhattan plot of differentially methylated CpGs in ALC vs. MF lambs. **D** The distribution of differentially methylated CpG sites on chromosome 4 and *SOSTDC1* gene. **E** Methylation pattern map of *SOSTDC1* gene was constructed based on the results of WGBS and prediction online tool (https://www.ebi.ac.uk/Tools/seqstats/emboss_cpgplot/)

ALC and MF siblings were used for whole genome resequencing (Additional file 2: Table S1) and WGBS (Additional file 4: Table S3). Phylogenic tree based on whole-genome SNPs showed that both ALC and MF wool sheep were clustered together with Australia Merino sheep, although they showed different wool types (Fig. 2A). This result indicated that the coarse wool phenotype of ALC lambs was not led by recent introgression hybridization of coarse wool sheep breed, but rather by some mechanism hidden in the population of fine wool sheep except for genomic DNA variation. Therefore, WGBS was conducted to identify epigenetic signals. PCA of WGBS data showed a distinct separation between ALC and MF wool lambs (Fig. 2B). A total of 1,586 differentially methylated regions (DMRs) related genes were screened, among which, 856 hypermethylated and 730 hypomethylated genes were identified (Additional file 5: Fig. S2). Manhattan plot was constructed by comparing the significance of differentially methylated CpG sites in different chromosomes, and the most significant differentially methylated CpG sites were located on chromosome 4 (Fig. 2C and D). Furthermore, all the top differentially methylated CpG sites were located on the second exon of the *SOSTDC1* gene (Fig. 2D). In addition, the gene structural analysis showed that the second exon of *SOSTDC1* gene has the highest GC content and unique CpG island (Fig. 2E), which was in line with the results of WGBS (Fig. 2D).

### Transcriptome analyses revealed the key pathway of ALC wool type

**Fig. 3:**
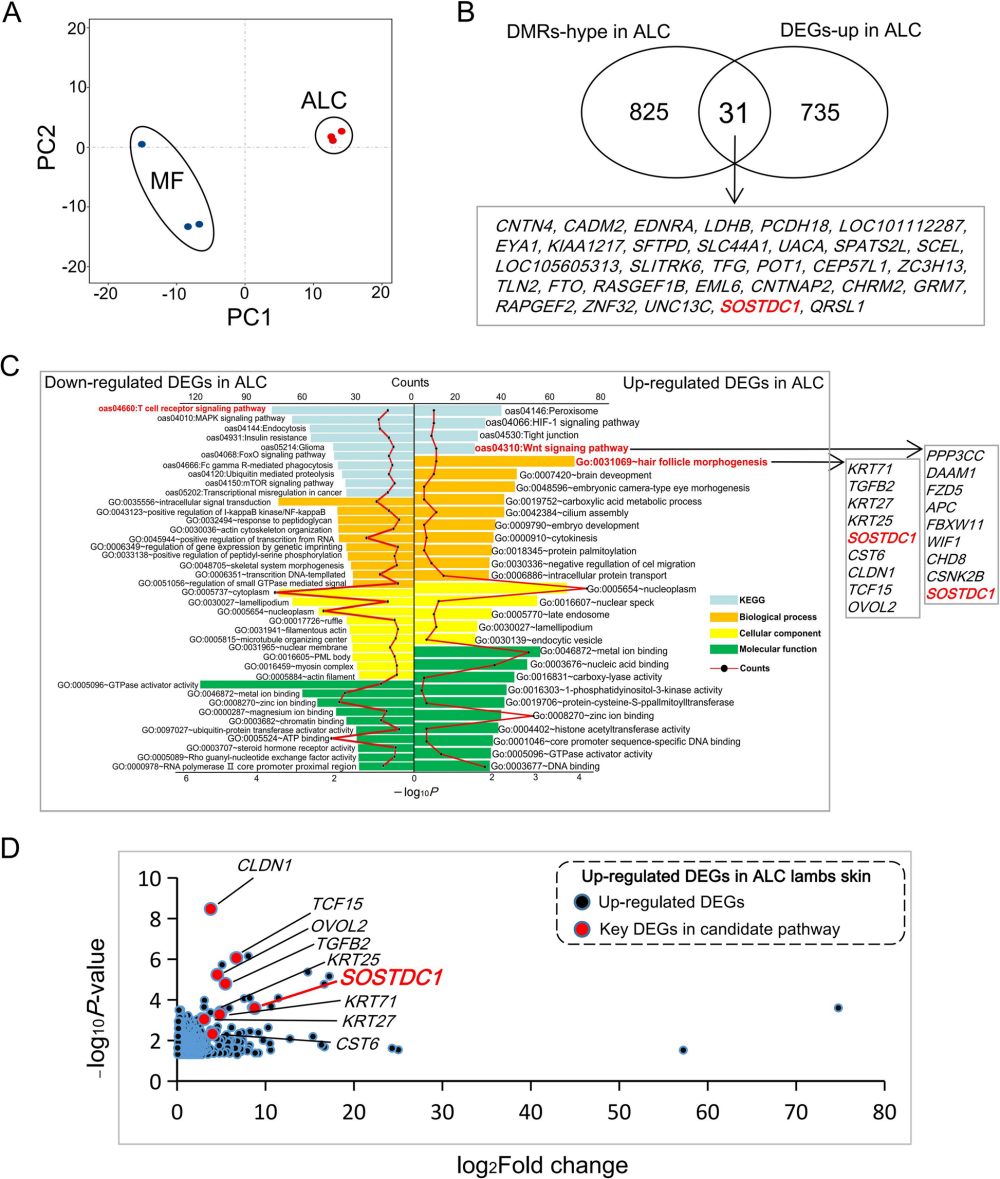
Transcriptomic data revealed the key signals of ALC wool trait. **A** PCA of RNA-seq data in ALC and MF wool lambs. **B** The overlapped genes between hypermethylated genes and up-regulated DEGs in ALC lambs. **C** Gene Ontology and KEGG pathway analysis of DEGs in ALC and MF wool lamb skin. The bottom *X*-axis represents the negative logarithm of the *P*-values (classic Fisher *t*-test) enriched by the corresponding gene ontology terms and KEGG pathways; The *X*-axis at the top shows the count level, also shown in red broken lines. **D** The expression of key candidate genes in ALC wool lamb skin

The same samples as for WGBS were used for RNA-seq to detect ALC related DEGs and corresponding pathways. PCA of RNA-seq data showed distinct expression signatures between ALC and MF skin tissues (Fig. 3A). A total of 728 genes were higher expressed in ALC lamb skin tissue and 805 genes were lower expressed in this group (Additional file 6: Table S4). *SOSTDC1* genes was found in the overlapping genes with up-regulated DEGs and hypermethylation related genes in ALC lambs (Fig. 3B). Among the up-regulated genes, “Hair follicle morphogenesis” was the first significantly highly enriched and obviously, this category serves as the most important symbol in hair follicle development (Fig. 3C). And the significantly enriched Wnt signaling pathway is also known for hair follicle morphogenesis (Fig. 3C) [29]. Notably, both of these pathways contain the *SOSTDC1* gene, which, along with other important genes, was at the top of all up-regulated DEGs in the skin of ALC lambs (Fig. 3D). Interestingly, “regulation of gene expression by genetic imprinting” was enriched in top ranking terms, which was consisted of four genes: *DNMT3A*, *ARID4A*, *BRCA1* and *CTCF*, all of which are related to the DNA methylation (Fig. 3C) [30–33].

### Analogy between ALC lambs and the modern coarse wool sheep breeds

**Fig. 4:**
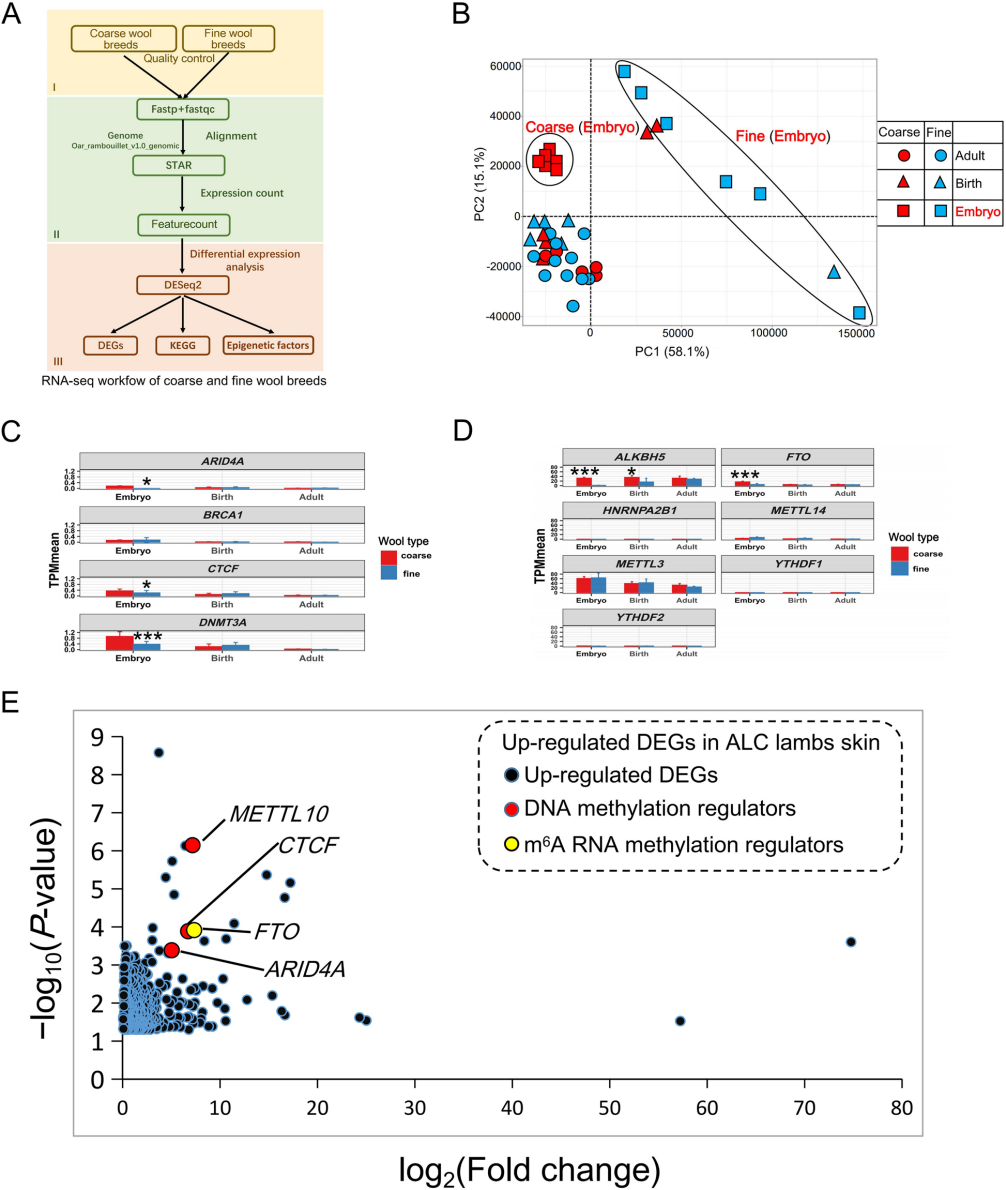
Analysis of differentially expressed genes at different developmental stages of skin tissue between coarse and fine wool breeds using transcriptome public data. **A** RNA-seq data analysis workflow of coarse and fine wool breeds. (I) Fastq data check and quality control. (II) Reads alignment and count. (III) Gene differential expression analysis. **B** PCA analysis of all expressed genes for sheep of different breeds and different periods. **C** The bar-plot of TPM mean for *DNMT3A*, *ARID4A*, *BRCA1*, *CTCF* gene between coarse and fine wool breeds at three stages. ^*^0.01 < *P* < 0.05, ^***^P < 0.001. **D** The bar-plot of TPM mean for *METTL3*, *METTL14*, *FTO*, *ALKBH5*, *YTHDF1*, *YTHDF2* and *HNRNPA2B1* gene between coarse and fine wool breed at three stages. ^*^0.01 < *P* < 0.05, ^***^P < 0.001. **E** The key epigenetic regulators expression in ALC wool lamb skin

**Fig. 5:**
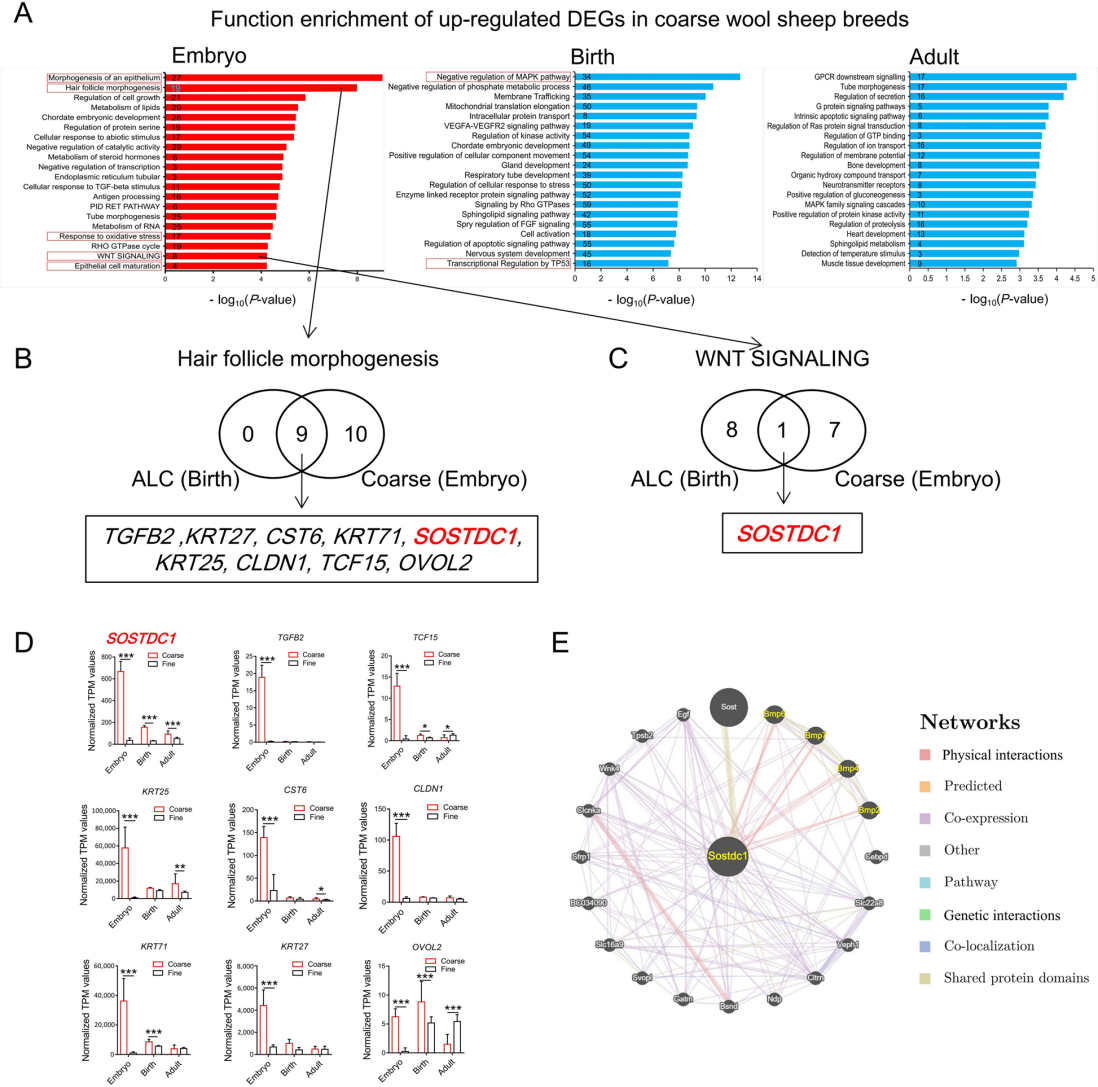
Comparison of the molecular mechanism of coarse wool follicle morphogenesis between ALC and coarse wool lamb breeds. **A** Analysis of top GO terms and KEGG pathways (Metascape database: http://www.metascape.org/) by using up and down-regulated differentially expressed genes in ALC wool lamb skin. −log10 of the *P* value as were taken at the *X*-axis for enrichment analysis. The up-regulated differentially expressed genes in embryos are shown in red bars, while the blue bars indicate the at-birth or adulthood stage. The numbers represent the number of genes enriched in each term. **B** The overlap of up-regulated DEGs in hair follicle morphogenesis process between ALC wool lambs and coarse wool lamb breeds. **C** The overlap of up-regulated DEGs in Wnt signal pathway between ALC wool lambs and coarse wool lamb breeds. **D** Expression analysis of candidate genes among coarse and fine wool breeds at different developmental stages. ^*^0.01 < *P* < 0.05, ^**^P < 0.01, ^***^P < 0.001. **E** Analysis of key protein interactions network by using online tool (GeneMANIA: http://genemania.org/)

In order to trace the significance of the ALC wool lamb model for study of wool follicle morphogenesis, we picked out the DEGs from the public transcriptome data of coarse and fine wool sheep breeds at three different developmental stages, of which are embryo, birth and adult (Fig. 4A and Additional file 3: Table S2). The raw expression counts from different sources were normalized using TPM method (Additional file 7: Fig. S3A). PCA analysis of the transcriptome data showed that coarse and fine wool sheep are virtually indistinguishable in adulthood, and there were no absolute differences between coarse and fine wool sheep at birth (Fig. 4B). However, embryonic coarse wool sheep were completely clustered together and clearly separated from embryonic fine wool sheep (Fig. 4B). Similar hair follicle morphogenesis related pathways were enriched in embryonic period of coarse wool breeds versus ALC wool lambs (Fig. 5A and Additional file 7: Fig. S3B). Moreover, similar significantly higher expression of epigenetic regulators was present in embryo of coarse wool sheep as in ALC lamb skin tissue (Fig. 4C and D), and ranked among the top of all up-regulated DEGs (Fig. 4E). This illustrated that the ALC lambs identified in this study have a comparable regulation pattern of wool follicle morphogenesis at the embryonic stage in the general coarse wool breeds, which provides better chance for investigating of genes and epigenetic regulatory mechanisms involved in wool type variations.

Both “Hair follicle morphogenesis” and “Wnt signal pathway” were enriched at the embryonic stage of coarse wool sheep breeds as well as in ALC lambs. In addition, all up-regulated DEGs related to hair follicle morphogenesis process in ALC group were overlapped to that in embryonic stage of the coarse wool sheep breeds (Fig. 5A–C). The candidate genes, including the *SOSTDC1*, showed significantly higher expression levels in coarse wool sheep skin tissues, and there is a decreasing expression trend from embryonic to birth and then to adulthood (Fig. 5D), indicating that these genes mainly function at the embryonic stage. Gene interaction analysis showed that the *SOSTDC1* gene has the potential to interoperate with BMP family proteins (Fig. 5E).

### Validation of *SOSTDC1* as a key candidate gene for wool follicle development

**Fig. 6:**
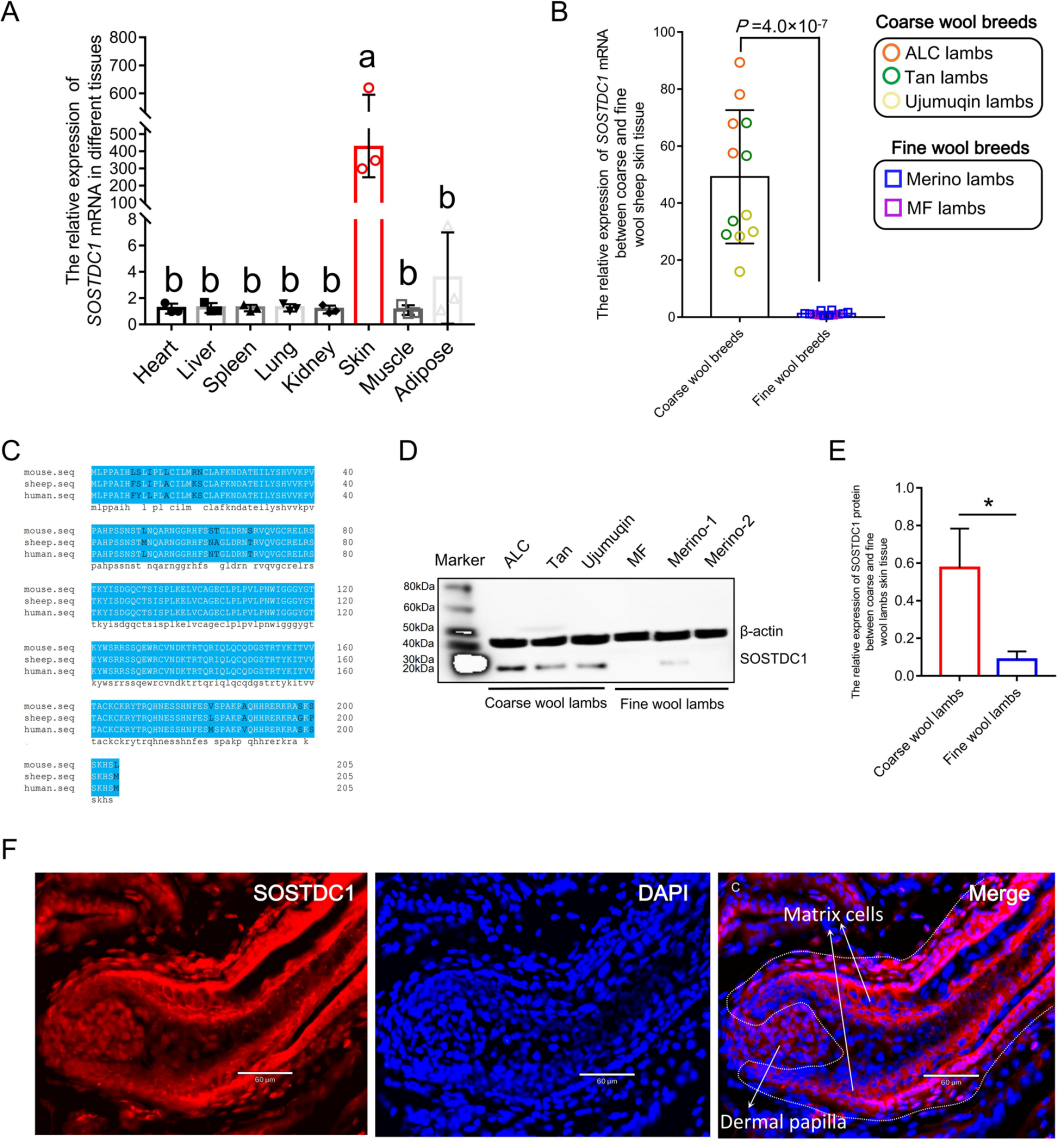
The expression analysis of *SOSTDC1* gene. **A** The relative expression of *SOSTDC1* at different tissues of newborn stage lambs, *n* = 3, the different letters indicate significant difference among tissues (*P* < 0.05). **B** The relative expression of *SOSTDC1* mRNA between coarse and fine wool lambs skin tissue, ALC lambs: the ancestral-like coarse lambs in this study (*n* = 4), Tan lambs: Tan sheep breed at one month of age (*n* = 4), Ujimqin lambs: Ujimqin sheep breed at one month of age (*n* = 4), Merino lambs: Aohan fine wool sheep at one month of age (*n* = 4), MF lambs: The half/full siblings of ALC lambs with fine wool (*n* = 4). **C** The amino acid sequence alignment of SOSTDC1 protein in mice, sheep and humans using DANMAN software. **D** Western-blot analysis of SOSTDC1 protein skin tissues of coarse and fine wool lambs. β-actin was used as housekeeping protein. ALC: the ancestral-like coarse lamb, Tan: Tan lamb, Ujimqin: Ujimqin lamb, MF: The half/full siblings of ALC lambs with fine wool, Merino-1 and Merino-2 indicate Aohan fine wool lamb. **E** Gray-scale analysis of Western-blot result in D using image J. ^*^0.01 < *P* < 0.05. **F **SOSTDC1 protein was detected in primary wool follicle of ALC lambs by immunofluorescence. The left, Immunofluorescent staining for *SOSTDC1*. The middle, Nuclei were stained with DAPI. The right, Merged immunofluorescence images using SOSTDC1 (red) and DAPI–stained nuclei (blue)

mRNA expression profile among lamb tissues showed that the *SOSTDC1* gene is specifically highly expressed in skin tissues and its expression was 274-fold higher than average in other tissues (Fig. 6A). Further analysis of the relative expression of the *SOSTDC1* gene in skin tissues between coarse and fine wool sheep at newborn stage showed that the expression of *SOSTDC1* gene in the coarse wool lambs was higher than in the fine wool lambs, with a highly significant difference at a multiplicity of 40 (Fig. 6B). Amino acid sequence alignment analysis showed that *SOSTDC1* protein has 93% homology among human, mouse and sheep (Fig. 6C). Therefore, the SOSTDC1 antibody was used for Western-blot assay, which showed that SOSTDC1 protein expression was significantly higher in coarse wool lambs than in fine wool lambs (Fig. 6D and E). In addition, localization of SOSTDC1 protein expression in skin tissues was detected by immunofluorescence, the result showed that the expression level of SOSTDC1 protein was higher in ALC wool lambs skin tissue than that of MF wool lambs, especially in the outer/inner root sheath of primary wool follicles (Additional file 8: Fig. S4). Besides, high magnification microscopic results of primary wool follicles of ALC wool lamb skin tissue showed that SOSTDC1 protein was highly expressed in the cell membrane of wool matrix cells and the nucleus of wool dermal papillae (the stem cells), respectively (Fig. 6F).

## Discussion

Primary wool follicles develop by embryonic d 50–90 (E50 to E90) in sheep and secondary wool follicles by embryonic d 80–100 [34]. In the Merino breed, the development of secondary follicles continues until three months after birth [35]. Larger primary wool follicles generate medullated coarse wool, and smaller primary and secondary wool follicles generate non-medullated fine wool [7]. In general, fine wool sheep are coated with non-medullated wool from birth [36]. The ALC wool lambs found in this study in a population of fine wool sheep with the same genetic background, but the ALC wool lambs retained significant medullated wool after birth. It was observed that its medullated wool gradually faded away with age, just as its late embryonic primary wool follicles gradually decreased [7]. Our study compared the embryos of modern coarse wool sheep and ALC wool lambs, which have distinct similarities in gene expression. The results of this study showed that Hair Follicle Morphogenesis and Wnt signaling pathways were enriched in both of the comparison groups. Moreover, all Hair Follicle Morphogenesis related genes of the ALC group also appeared in the same pathway of up-regulated DEGs in the embryonic stage of coarse wool breeds. This suggests that the molecular mechanisms of wool follicle morphogenesis are somewhat similar between ALC lambs and other coarse wool breeds. The ALC wool trait is likely due to the delay from the embryonic stage to the early postnatal stage in the process of primary wool follicle development. Although we found significantly associated genes, the related molecular mechanisms still need continued investigation.

Among the candidate genes, numerous studies have shown that *SOSTDC1* and *TGFB2* play crucial roles for normal hair follicle formation, for example, *SODTDC1* has an important impact on the size of primary hair placodes [37]. Protein interaction analysis has shown that the proteins interacting with SOSTDC1 are mainly members of the BMP family, such as BMP2, BMP4, BMP6 and BMP7 (Fig. 5E), and SOSTDC1 is an antagonist of the BMP signaling pathway, which inhibits its activity by binding to BMP family proteins [38]. It has also been shown that active BMP signaling has an inhibitory effect on hair follicle development [39, 40]. Therefore, it can be hypothesized that *SOSTDC1* is able to promote hair follicle development by inhibiting the activity of the BMP signaling pathway. In addition, it was also shown that *SOSTDC1* and *TGFB2* genes are critical for the regulation of the immune system, with *SOSTDC1* secreted from dermal lymphatic vessels identified as growth a factor for hair follicle growth [41]. Moreover, three TGF-β isoforms (*TGFB1* [42], *TGFB2* [43] and *TGFB3* [44]) are expressed in mammalian epithelium, they all belong to the TGF signaling pathway [45], which are all closely associated with immune regulation [46] and hair follicle development [47]. Besides, *TGFB3* can be secreted by regulatory T cells and promotes hair follicle regeneration [48], suggesting a close association between the immune system and hair follicle development. In fact, numerous studies have shown that regulatory T cells in the skin play a major role in hair follicle biology [49–51]. In this study, the down-regulated DEGs of ALC group were also enriched to the immune signaling pathway and ranked first in the list (Fig. 3C). This result further suggested the negative correlation between immunity and wool follicle development [49]. In fact, it has been shown that the highly expressed genes at E135-P30 were significantly enriched in various immune processes [11], and this is the time when the development of primary wool follicles are inhibited while secondary wool follicles are growing vigorously [34]. Therefore, in the ALC model, we hypothesize that *SOSTDC1* and *TGFB2* likely work together as a bridge to maintain the balance between immunity and wool follicle development, which is also expected further explorations.

When analyzing the DEGs of wool type between the ALC/MF model and coarse/fine wool breeds, we found that numerous epigenetic regulators were significantly differentially expressed between the coarse and fine wool types at the key stage for wool follicle morphogenesis. This suggests that the wool types are likely to be epigenetically regulated [14]. In this study, we found that the differential methylation signal of *SOSTDC1* gene was ranked at the top, and combined with the expression characteristics of *SOSTDC1* gene, we suggest that differential methylation of *SOSTDC1* is the main reason for differential expression of the *SOSTDC1* gene, and consequently the formation of the ALC wool type. Indeed, in the studies related to genome-wide methylation analysis to date, the significantly differential methylation sites tend to be scattered across chromosomes [52–55], however, in this study, the differentially methylated sites were concentrated in one gene and the signals were much more significant than other sites in the genome. This result is most likely due to three reasons: first, the samples tested are siblings with a relatively consistent genetic background, second, the target characteristics are distinctly different and the time points and body parts where the samples were collected are precisely consistent among individuals, and finally, there does exist a CpG island on the second exon of sheep *SOSTDC1* gene (Fig. 2E) that is susceptible to modification by DNA methylation. In addition, differentially expressed RNA methylation regulators were also screened for significantly higher expression in skin tissues of ALC lambs and coarse wool sheep embryos (Fig. 4D and E). This suggests that RNA methylation may influence wool follicle morphogenesis [56, 57]. In our previous study, we found that PI3K/AKT signaling pathway related genes were affected by RNA methylation in the ALC group by meRIP-seq [58], therefore, we speculated that both DNA and RNA methylation contributes to the generation of the ALC wool type.

## Conclusions

In this study, we found newborn lambs with coarse medullated wool, which were so called ancestral-like coarse (ALC) wool sheep, in a modern fine (MF) wool sheep population by MOET breeding. Using this model, we found the strongest differential methylation locus located in the second exon of the *SOSTDC1* gene, which corresponds to the differential expression of the *SOSTDC1* gene revealed by transcriptome analysis. Validation experiments showed that *SOSTDC1* gene was specifically overexpressed in the nucleus of wool follicle stem cells of ALC wool lambs, indicating its importance in primary wool follicle development. This study provides a new perspective for the role of epigenetics in wool follicle development, and its role in wool sheep domestication and breeding.

## Supplementary Information

**Additional file 1: Fig. S1.** The genealogical relations of ALC wool type lambs.

**Additional file 2: Table S1.** Information about part of the whole genome re-sequence data in this study.

**Additional file 3: Table S2.** Information about part of the RNA-seq data in this study.

**Additional file 4: Table S3.** The evaluation of WGBS data.

**Additional file 5: Fig. S2.** The filtration of differentially methylated genes and the statistics of DMRs in different gene regulatory elements, Filtering criteria: −log_10_*P *> 20.

**Additional file 6: Table S4.** The list of DEGs between ALC and MF wool lambs.

**Additional file 7: Fig. S3.** Analysis of differentially expressed genes at different developmental stages of skin tissue between coarse and fine wool breeds using transcriptome public data. **A** The raw expression count was normalized using TPM method. **B** The heatmap of highly expressed DEGs in sheep skin of different breeds and different periods.

**Additional file 8: Fig. S4.** SOSTDC1 protein was detected in skin tissues of ALC and MF lambs by immunofluorescence. Nuclei were stained with DAPI.

## Data Availability

The WGS, WGBS-seq and RNA-seq data reported in this study had been deposited in National Center for Biotechnology Information with the accession numbers PRJNA760647, PRJNA760832 and PRJNA760789.

## Abbreviations

ALC: Ancestral-like coarse
BS-Seq: Bisulfite sequencing
DEGs: Differentially expressed genes
DMG: Differentially methylated gene
DMR: Differentially methylated region
FDR: False discovery rate
GO: Gene Ontology
KEGG: Kyoto Encyclopedia of Genes and Genomes
mC: 5-methylated cytosines
MF: Modern fine
MOET: Multiple ovulation and embryo transfer
PCR: Polymerase chain reaction
WGBS: Whole genome bisulfite sequencing
WGS: Whole-genome resequencing
