# Supplementary figures and images for: Genome-wide DNA methylation and transcriptome analyses reveal the key gene for wool type variation in sheep
Source: J Anim Sci Biotechnol. 2023 Jul 8;14:88. doi: 10.1186/s40104-023-00893-6 (PMC10329393; doi:10.1186/s40104-023-00893-6)

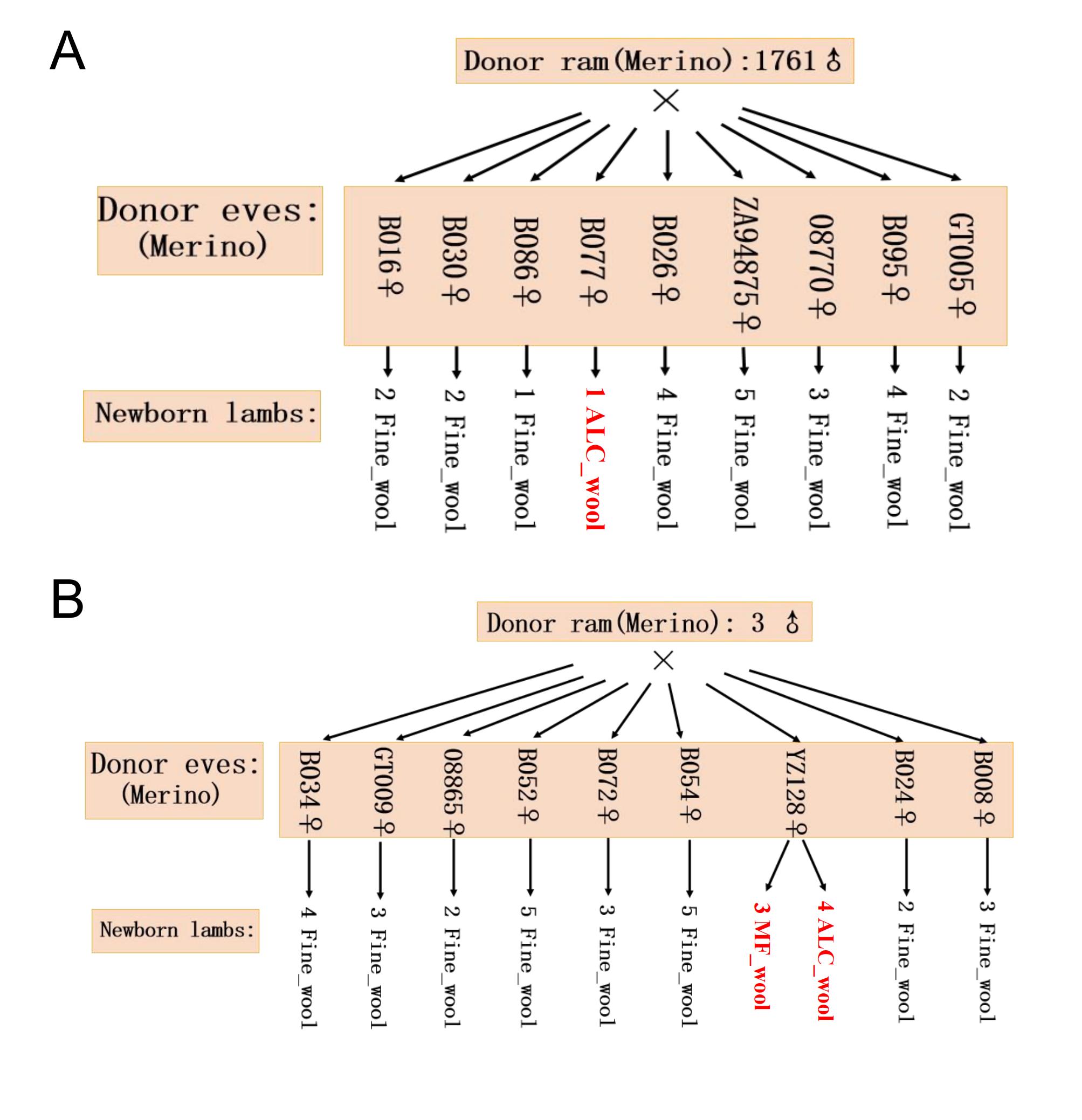

Supplement: Supplementary file 1 — Additional file 1: Fig. S1. The genealogical relations of ALC wool type lambs. [file 40104_2023_893_MOESM1_ESM.jpg]

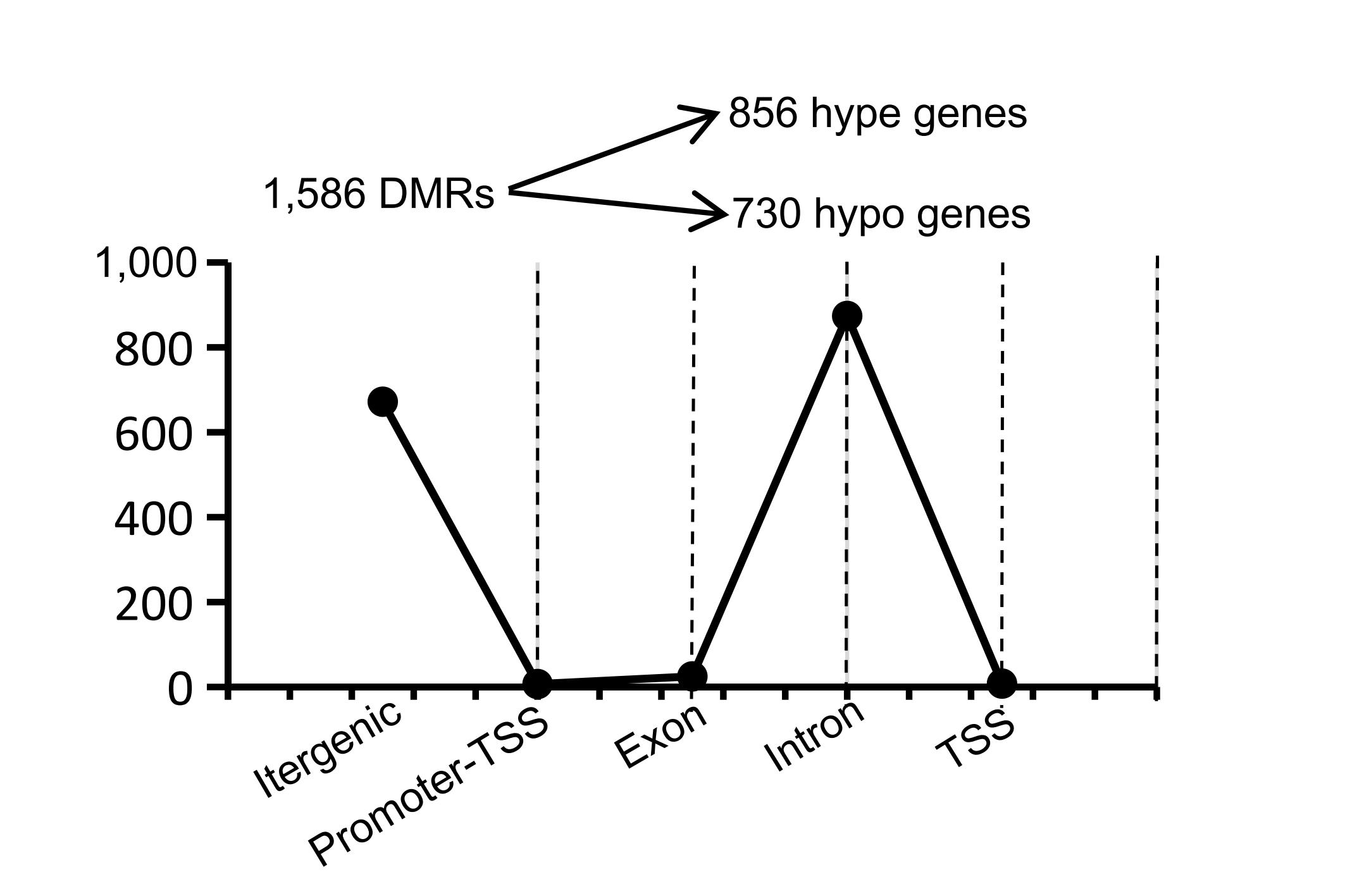

Supplement: Supplementary file 5 — Additional file 5: Fig. S2. The filtration of differentially methylated genes and the statistics of DMRs in different gene regulatory elements, Filtering criteria: −log10P > 20. [file 40104_2023_893_MOESM5_ESM.jpg]

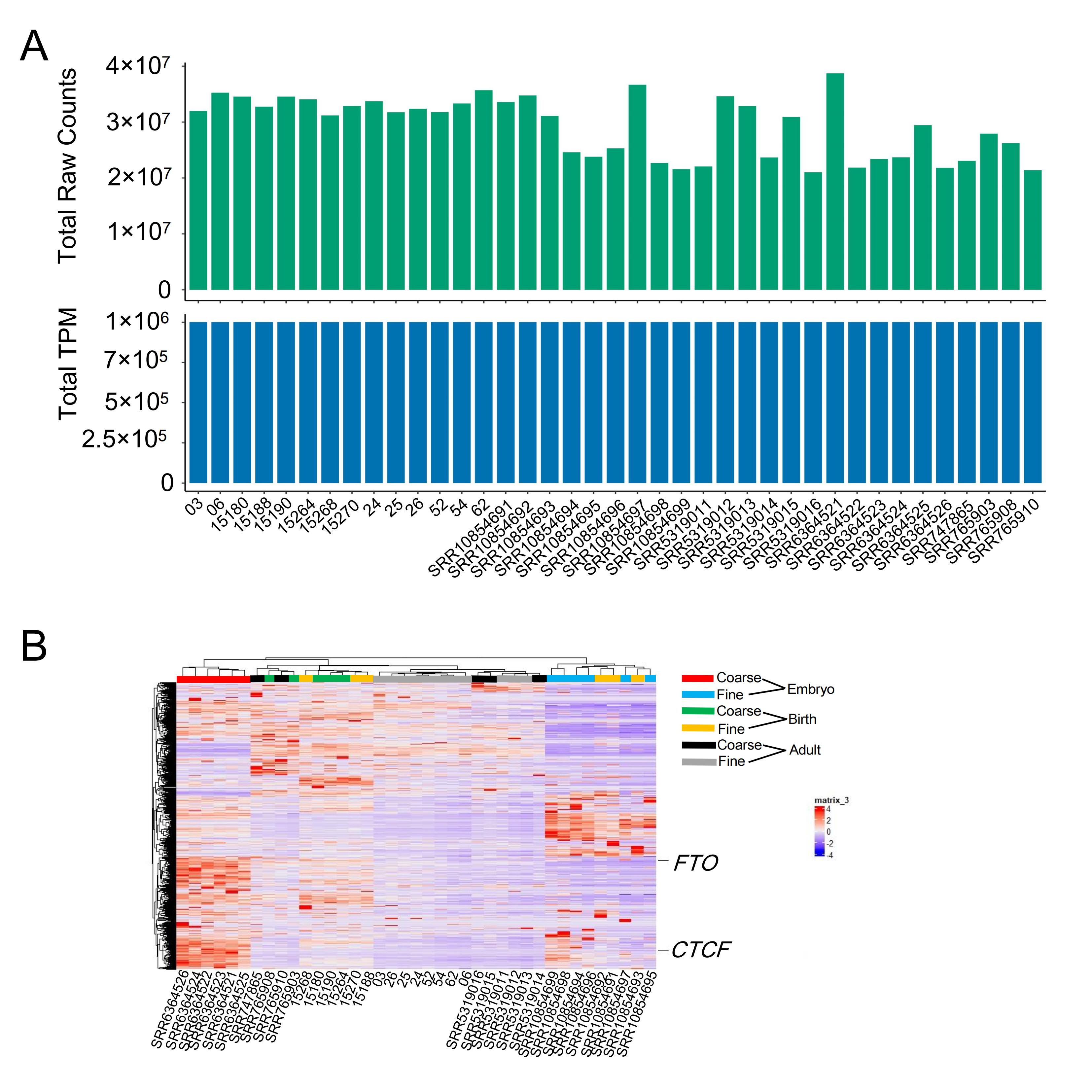

Supplement: Supplementary file 7 — Additional file 7: Fig. S3. Analysis of differentially expressed genes at different developmental stages of skin tissue between coarse and fine wool breeds using transcriptome public data. A The raw expression count was normalized using TPM method. B The heatmap of highly expressed DEGs in sheep skin of different breeds and different periods. [file 40104_2023_893_MOESM7_ESM.jpg]

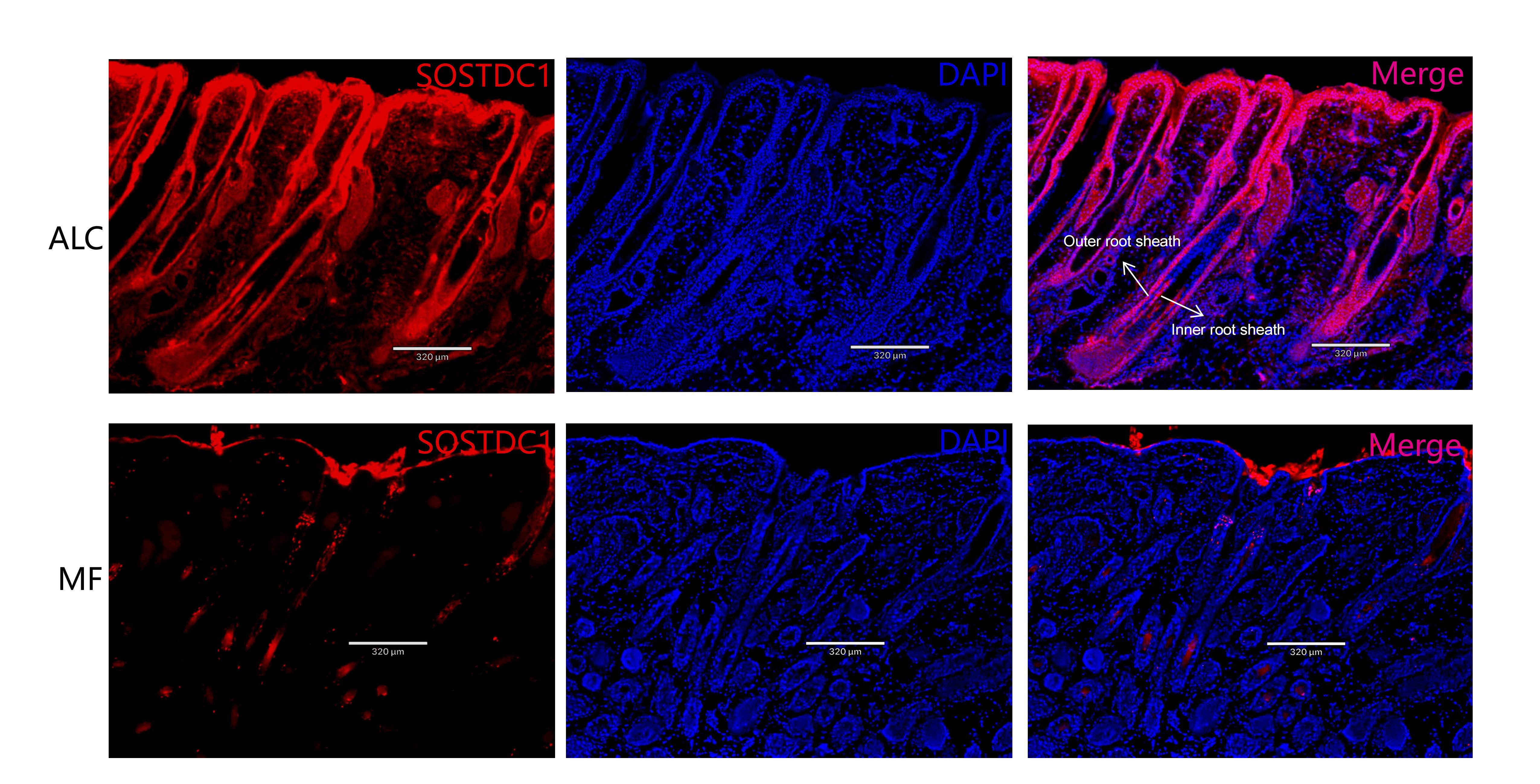

Supplement: Supplementary file 8 — Additional file 8: Fig. S4. SOSTDC1 protein was detected in skin tissues of ALC and MF lambs by immunofluorescence. Nuclei were stained with DAPI. [file 40104_2023_893_MOESM8_ESM.jpg]
